# Supplementary material for: Active site specificity profiling datasets of matrix metalloproteinases (MMPs) 1, 2, 3, 7, 8, 9, 12, 13 and 14
Source: Data Brief. 2016 Feb 22;7:299–310. doi: 10.1016/j.dib.2016.02.036 (PMC4777984; doi:10.1016/j.dib.2016.02.036)
Supplement: Supplementary file 10 — Supplementary material [file mmc10.zip › WebPICS_hMMP2_G_1%/PICS_results.html]

 

PICS results


- # About
- # Manual
- # Analysis
- # Results

- Redraw
- Seqlogo
- Dependency
- Coop

**PICS analysis of protease: hMMP2\_G\_1%**  
246 cleavage sites analyzed (total was 254)  
PICS library made with (T)rypsin, (G)luC or (C)hymotrypsin: G  

|  |  |
| --- | --- |
| Positional occurences    **Values > 10 % are shown** (table for total and relative (in %) values) | Occurences relative to natural abundance   **Values > 2 fold natural abundance are shown** (table) |
|  |  |

  
  
Results (including this html file) in one compressed file for local use.
  
  
Total occurences (open csv)

|  |  |  |  |  |  |  |  |  |  |  |  |  |
| --- | --- | --- | --- | --- | --- | --- | --- | --- | --- | --- | --- | --- |
|  | P6 | P5 | P4 | P3 | P2 | P1 | P1prime | P2prime | P3prime | P4prime | P5prime | P6prime |
| A | 28 | 17 | 16 | 33 | 70 | 34 | 12 | 9 | 53 | 25 | 22 | 18 |
| C | 4 | 1 | 4 | 2 | 2 | 10 | 8 | 3 | 0 | 2 | 2 | 1 |
| D | 18 | 13 | 14 | 1 | 3 | 22 | 1 | 2 | 9 | 10 | 9 | 19 |
| E | 1 | 1 | 4 | 0 | 0 | 0 | 1 | 0 | 0 | 1 | 2 | 6 |
| F | 6 | 7 | 9 | 2 | 5 | 3 | 9 | 15 | 2 | 9 | 11 | 5 |
| G | 16 | 25 | 15 | 3 | 24 | 37 | 4 | 2 | 37 | 20 | 14 | 25 |
| H | 3 | 4 | 3 | 2 | 4 | 7 | 0 | 8 | 2 | 6 | 4 | 8 |
| I | 7 | 9 | 11 | 33 | 2 | 2 | 32 | 35 | 6 | 10 | 14 | 21 |
| K | 11 | 9 | 14 | 7 | 13 | 9 | 2 | 31 | 18 | 16 | 18 | 22 |
| L | 6 | 11 | 16 | 26 | 9 | 5 | 120 | 29 | 10 | 30 | 35 | 25 |
| M | 5 | 7 | 1 | 5 | 1 | 6 | 10 | 7 | 4 | 4 | 3 | 4 |
| N | 5 | 9 | 10 | 3 | 8 | 23 | 0 | 10 | 18 | 12 | 4 | 12 |
| P | 9 | 12 | 13 | 54 | 4 | 9 | 2 | 2 | 3 | 15 | 12 | 20 |
| Q | 6 | 14 | 12 | 7 | 18 | 11 | 8 | 14 | 5 | 26 | 21 | 11 |
| R | 4 | 4 | 4 | 1 | 12 | 10 | 0 | 10 | 1 | 9 | 14 | 7 |
| S | 12 | 11 | 16 | 7 | 37 | 30 | 4 | 11 | 38 | 18 | 17 | 8 |
| T | 10 | 8 | 16 | 8 | 7 | 10 | 1 | 20 | 11 | 12 | 13 | 15 |
| V | 8 | 22 | 23 | 29 | 7 | 2 | 18 | 29 | 27 | 20 | 28 | 14 |
| W | 1 | 0 | 0 | 1 | 0 | 4 | 5 | 1 | 0 | 0 | 0 | 1 |
| Y | 0 | 5 | 8 | 2 | 3 | 3 | 9 | 8 | 2 | 1 | 3 | 4 |

  
  
Relative occurences in % (open csv)

|  |  |  |  |  |  |  |  |  |  |  |  |  |
| --- | --- | --- | --- | --- | --- | --- | --- | --- | --- | --- | --- | --- |
|  | P6 | P5 | P4 | P3 | P2 | P1 | P1prime | P2prime | P3prime | P4prime | P5prime | P6prime |
| A | 11.4 | 6.9 | 6.5 | 13.4 | 28.5 | 13.8 | 4.9 | 3.7 | 21.5 | 10.2 | 8.9 | 7.3 |
| C | 1.6 | 0.4 | 1.6 | 0.8 | 0.8 | 4.1 | 3.3 | 1.2 | 0.0 | 0.8 | 0.8 | 0.4 |
| D | 7.3 | 5.3 | 5.7 | 0.4 | 1.2 | 8.9 | 0.4 | 0.8 | 3.7 | 4.1 | 3.7 | 7.7 |
| E | 0.4 | 0.4 | 1.6 | 0.0 | 0.0 | 0.0 | 0.4 | 0.0 | 0.0 | 0.4 | 0.8 | 2.4 |
| F | 2.4 | 2.8 | 3.7 | 0.8 | 2.0 | 1.2 | 3.7 | 6.1 | 0.8 | 3.7 | 4.5 | 2.0 |
| G | 6.5 | 10.2 | 6.1 | 1.2 | 9.8 | 15.0 | 1.6 | 0.8 | 15.0 | 8.1 | 5.7 | 10.2 |
| H | 1.2 | 1.6 | 1.2 | 0.8 | 1.6 | 2.8 | 0.0 | 3.3 | 0.8 | 2.4 | 1.6 | 3.3 |
| I | 2.8 | 3.7 | 4.5 | 13.4 | 0.8 | 0.8 | 13.0 | 14.2 | 2.4 | 4.1 | 5.7 | 8.5 |
| K | 4.5 | 3.7 | 5.7 | 2.8 | 5.3 | 3.7 | 0.8 | 12.6 | 7.3 | 6.5 | 7.3 | 8.9 |
| L | 2.4 | 4.5 | 6.5 | 10.6 | 3.7 | 2.0 | 48.8 | 11.8 | 4.1 | 12.2 | 14.2 | 10.2 |
| M | 2.0 | 2.8 | 0.4 | 2.0 | 0.4 | 2.4 | 4.1 | 2.8 | 1.6 | 1.6 | 1.2 | 1.6 |
| N | 2.0 | 3.7 | 4.1 | 1.2 | 3.3 | 9.3 | 0.0 | 4.1 | 7.3 | 4.9 | 1.6 | 4.9 |
| P | 3.7 | 4.9 | 5.3 | 22.0 | 1.6 | 3.7 | 0.8 | 0.8 | 1.2 | 6.1 | 4.9 | 8.1 |
| Q | 2.4 | 5.7 | 4.9 | 2.8 | 7.3 | 4.5 | 3.3 | 5.7 | 2.0 | 10.6 | 8.5 | 4.5 |
| R | 1.6 | 1.6 | 1.6 | 0.4 | 4.9 | 4.1 | 0.0 | 4.1 | 0.4 | 3.7 | 5.7 | 2.8 |
| S | 4.9 | 4.5 | 6.5 | 2.8 | 15.0 | 12.2 | 1.6 | 4.5 | 15.4 | 7.3 | 6.9 | 3.3 |
| T | 4.1 | 3.3 | 6.5 | 3.3 | 2.8 | 4.1 | 0.4 | 8.1 | 4.5 | 4.9 | 5.3 | 6.1 |
| V | 3.3 | 8.9 | 9.3 | 11.8 | 2.8 | 0.8 | 7.3 | 11.8 | 11.0 | 8.1 | 11.4 | 5.7 |
| W | 0.4 | 0.0 | 0.0 | 0.4 | 0.0 | 1.6 | 2.0 | 0.4 | 0.0 | 0.0 | 0.0 | 0.4 |
| Y | 0.0 | 2.0 | 3.3 | 0.8 | 1.2 | 1.2 | 3.7 | 3.3 | 0.8 | 0.4 | 1.2 | 1.6 |

  
  
Occurences relative to natural abundance   

|  |  |  |  |  |  |  |  |  |  |  |  |  |
| --- | --- | --- | --- | --- | --- | --- | --- | --- | --- | --- | --- | --- |
|  | P6 | P5 | P4 | P3 | P2 | P1 | P1prime | P2prime | P3prime | P4prime | P5prime | P6prime |
| A | 1.4 | 0.8 | 0.8 | 1.6 | 3.4 | 1.7 | 0.6 | 0.4 | 2.6 | 1.2 | 1.1 | 0.9 |
| C | 1.2 | 0.3 | 1.2 | 0.6 | 0.6 | 3.0 | 2.4 | 0.9 | 0.0 | 0.6 | 0.6 | 0.3 |
| D | 1.3 | 1.0 | 1.0 | 0.1 | 0.2 | 1.6 | 0.1 | 0.1 | 0.7 | 0.8 | 0.7 | 1.4 |
| E | 0.1 | 0.1 | 0.2 | 0.0 | 0.0 | 0.0 | 0.1 | 0.0 | 0.0 | 0.1 | 0.1 | 0.4 |
| F | 0.6 | 0.7 | 1.0 | 0.2 | 0.5 | 0.3 | 1.0 | 1.6 | 0.2 | 1.0 | 1.2 | 0.5 |
| G | 0.9 | 1.4 | 0.9 | 0.2 | 1.4 | 2.1 | 0.2 | 0.1 | 2.1 | 1.1 | 0.8 | 1.4 |
| H | 0.5 | 0.7 | 0.5 | 0.4 | 0.7 | 1.2 | 0.0 | 1.5 | 0.4 | 1.1 | 0.7 | 1.5 |
| I | 0.5 | 0.6 | 0.8 | 2.2 | 0.1 | 0.1 | 2.2 | 2.4 | 0.4 | 0.7 | 1.0 | 1.4 |
| K | 0.8 | 0.6 | 1.0 | 0.5 | 0.9 | 0.6 | 0.1 | 2.2 | 1.2 | 1.1 | 1.2 | 1.5 |
| L | 0.2 | 0.5 | 0.7 | 1.1 | 0.4 | 0.2 | 5.0 | 1.2 | 0.4 | 1.3 | 1.5 | 1.1 |
| M | 0.8 | 1.2 | 0.2 | 0.8 | 0.2 | 1.0 | 1.7 | 1.2 | 0.7 | 0.7 | 0.5 | 0.7 |
| N | 0.5 | 0.9 | 1.0 | 0.3 | 0.8 | 2.3 | 0.0 | 1.0 | 1.8 | 1.2 | 0.4 | 1.2 |
| P | 0.8 | 1.0 | 1.1 | 4.7 | 0.3 | 0.8 | 0.2 | 0.2 | 0.3 | 1.3 | 1.0 | 1.7 |
| Q | 0.6 | 1.4 | 1.2 | 0.7 | 1.9 | 1.1 | 0.8 | 1.4 | 0.5 | 2.7 | 2.2 | 1.1 |
| R | 0.3 | 0.3 | 0.3 | 0.1 | 0.9 | 0.7 | 0.0 | 0.7 | 0.1 | 0.7 | 1.0 | 0.5 |
| S | 0.8 | 0.7 | 1.0 | 0.4 | 2.3 | 1.9 | 0.2 | 0.7 | 2.4 | 1.1 | 1.1 | 0.5 |
| T | 0.8 | 0.6 | 1.2 | 0.6 | 0.5 | 0.8 | 0.1 | 1.5 | 0.8 | 0.9 | 1.0 | 1.1 |
| V | 0.5 | 1.3 | 1.4 | 1.7 | 0.4 | 0.1 | 1.1 | 1.7 | 1.6 | 1.2 | 1.7 | 0.8 |
| W | 0.4 | 0.0 | 0.0 | 0.4 | 0.0 | 1.5 | 1.9 | 0.4 | 0.0 | 0.0 | 0.0 | 0.4 |
| Y | 0.0 | 0.7 | 1.1 | 0.3 | 0.4 | 0.4 | 1.3 | 1.1 | 0.3 | 0.1 | 0.4 | 0.5 |
